# Postprandial Oxidative Stress and Gastrointestinal Hormones: Is There a Link?

**DOI:** 10.1371/journal.pone.0103565

**Published:** 2014-08-20

**Authors:** Hana Malinska, Hana Kahleova, Ondrej Topolcan, Jindra Vrzalova, Olena Oliyarnyk, Ludmila Kazdova, Lenka Belinova, Martin Hill, Terezie Pelikanova

**Affiliations:** 1 Institute for Clinical and Experimental Medicine, Prague, Czech Republic; 2 Faculty Hospital Pilsen, Pilsen, Czech Republic; 3 Institute of Endocrinology, Prague, Czech Republic; 4 Charles University, 1st Faculty of Medicine, Prague, Czech Republic; Monash University, Australia

## Abstract

**Background:**

Abnormal postprandial elevation of plasma glucose and lipids plays an important role in the pathogenesis of diabetes and strongly predicts cardiovascular mortality. In patients suffering from type 2 diabetes (T2D) postprandial state is associated with oxidative stress, cardiovascular risk and, probably, with impairment of both secretion and the effect of gastrointestinal peptides. Evaluating postprandial changes of gastrointestinal hormones together with changes in oxidative stress markers may help to understand the mechanisms behind the postprandial state in diabetes as well as suggest new preventive and therapeutical strategies.

**Methods:**

A standard meal test has been used for monitoring the postprandial concentrations of gastrointestinal hormones and oxidative stress markers in patients with T2D (n = 50) compared to healthy controls (n = 50). Blood samples were drawn 0, 30, 60, 120 and 180 minutes after the standard meal.

**Results:**

Both basal and postprandial plasma concentrations of glucose and insulin proved to be significantly higher in patients with T2D, whereas plasma concentrations of ghrelin showed significantly lower values during the whole meal test. In comparison with healthy controls, both basal and postprandial concentrations of almost all other gastrointestinal hormones and lipoperoxidation were significantly increased while ascorbic acid, reduced glutathione and superoxide dismutase activity were decreased in patients with T2D. A positive relationship was found between changes in GIP and those of glucose and immunoreactive insulin in diabetic patients (p<0.001 and p<0.001, respectively) and between changes in PYY and those of glucose (p<0.01). There was a positive correlation between changes in GIP and PYY and changes in ascorbic acid in patients with T2D (p<0.05 and p<0.001, respectively).

**Conclusion/Interpretation:**

Apart from a positive relationship of postprandial changes in GIP and PYY with changes in ascorbic acid, there was no direct link observed between gastrointestinal hormones and oxidative stress markers in diabetic patients.

**Trial Registration:**

ClinicalTrials.gov NCT01572402

## Introduction

The postprandial dysmetabolism plays an important role in the pathogenesis of type 2 diabetes (T2D) and its complications. Abnormal postprandial elevation of plasma glucose and lipids is closely tied to insulin resistance and may occur in the absence of overt T2D. Postmeal hyperglycemia and hyperlipidemia increases the risk of cardiovascular diseases in diabetic patients and may predict cardiovascular risk more strongly than fasting values or even long-term parameters such as glycated hemoglobin [1].

In patients with T2D, acute hyperglycemia and hypertriglyceridemia lead to endothelial dysfunction, induce oxidative stress, increase the inflammatory milieu, affect coagulation, and, probably, impair secretion and diminish effect of gastrointestinal peptides [2].

Incretin hormones, which are released from the gastrointestinal tract in response to nutrient ingestion to enhance glucose-dependant insulin secretion, aid the overall maintenance of glucose homeostasis through slowing of gastric emptying, inhibition of glucagon secretion and control of body weight [3]. Two incretins - glucagon-like peptide-1 (GLP-1) (which has received the most pharmacological attention), and gastric inhibitory peptide (GIP) - were found to exert major glucoregulatory actions [4]. The impaired incretin effect may contribute to delayed and attenuated insulin response during a meal in T2D [5], [6], [7]. The mechanism which would make clear the diminished effect of gastrointestinal hormones in patients with T2D is not completely understood. It is not clear whether the loss of incretin secretion is a cause or rather a consequence of hyperglycaemia.

Appetite hormones, ghrelin and leptin, are also known to play a prominent role in glucose homeostasis and the regulation of energy. Changes in plasma concentrations of ghrelin and leptin in diabetic patients are strongly associated with hyperinsulinemia and are probably of great importance for the pathogenesis of diabetes [8].

According to recent studies, oxidative stress is supposed to be the link between acute postprandial hyperglycemia and cardiovascular risk in patients with T2D [9]. In some studies, several markers of oxidative damage such as TBARS [10], isoprostanes [11] and protein carbonyls [12] have been found to increase 2–3 hours after an oral glucose load (OGTT). However, there is still lack of information about the relationship of oxidative stress, gastrointestinal and appetite hormones, particularly during the postmeal phase.

Evaluating the effect of gastrointestinal hormones together with changes in oxidative stress markers may contribute to better understanding of the mechanisms underlying the postprandial state in patients suffering from T2D and thus suggest new preventive and therapeutical strategies. A standard meal test was used for monitoring the postprandial concentrations of gastrointestinal hormones and oxidative stress markers in patients with T2D compared to healthy controls. To the best knowledge of the authors, they are the first ones to try to find a link between postprandial oxidative stress and gastrointestinal hormones in a clinical and physiological setting.

## Materials and Methods

### Study subjects and design

The study group consisted of 50 patients with T2D and 50 healthy controls. Their characteristics are featured in Table 1. The mean age was 55 years, approximately 50% of the subjects were men, the mean duration of diabetes in diabetic subjects was 9.8 years. The study protocol was approved by the Ethics Committee of the Thomayer Hospital and Institute for Clinical and Experimental Medicine in Prague, Czech Republic. All participants have signed a written informed consent. Clinical Trial.gov number, NCT01572402. The protocol for this trial and supporting CONSORT checklist are available as supporting information; see checklist S1 and Protocol S1.

**Table 1:** General characteristics of the Diabetic and Control Population.

Characteristics	Diabetics (n = 50)	Controls (n = 50)
Age – years	56±6	54±8
Male - No. (%)	23 (46)	23 (46)
Female - No. (%)	27 (54)	27 (54)
Smokers – No. (%)	11 (22)	7 (14)
Weight – kg	97±17	71±11
BMI – kg.m^−2^	33.3±5.6	24.4±2.5
Waist – cm	107±13	85±8
Hips – cm	115±12	98±5
HbA1c (DCCT) – %	7.0±3.2	5.6±2.4
HbA1c (IFCC) – mmol/mol	53.7±12.0	37.3±2.7
Fasting glucose level – mmol/l	8.0±3.1	5.0±0.4
Duration of diabetes – years	9.8±6.3	

Data are means ± SD.

Eligibility criteria for participants were set as following: age 30 to 70 years, both genders. Inclusion criteria – diabetes duration at least 1 year, BMI 27–50 kg/m^2^, exclusion criteria – insulin therapy.

### Procedures

All measurements were taken on an outpatient basis, after 10-h to 12-h overnight fasting with only tap water allowed ad libitum. In this single-center study the samples were collected at the Laboratory of Clinical Pathophysiology in Institute for Clinical and Experimental Medicine.

#### Standard meal tests

Posprandial state was tested after stimulation with a standard breakfast (The Baguette Cheese Gourmet produced by Crocodille, 453 kcal, 45% carbohydrates, 17% proteins, 38% lipids). This is the part of the randomised clinical study, where we observed the postprandial effect after three different sandwiches in the random order in patients with T2D and healthy controls. Presented data relate to the cheese sandwich only. The nurses engaged in the study generated the random sequence of the meals and assigned participants to interventions. Neither the study staff nor the participants could be blinded to the content of the meals. The participants ate the sandwiches in the laboratory under the observation by nurses.

Plasma glucose, immunoreactive insulin, C-peptide, triglycerides, free fatty acids, oxidative stress markers and gastrointestinal hormones were all measured after 0, 30, 60, 120, and 180 minutes.

#### Analytic methods

Blood samples were drawn in the fasting state and then 30, 60, 120 and 180 minutes after the standard meal. Protease and Dipeptidyl peptidase-4 inhibitors were added into two samples at each time point. Plasma glucose was analysed using the Beckman Analyzer glucose-oxidase method (Beckman Instruments Inc., Fullerton, CA, USA). Serum immunoreactive insulin and C-peptide concentrations were determined using Insulin and C-peptide IRMA kits (Immunotech, Prague, Czech Republic). Plasma lipids were measured using enzymatic methods (Roche, Basel, Switzerland).

Gastrointestinal and appetite hormones: Concentrations of GLP-1, GIP, amylin, pancreatic polypeptide (PP), peptide YY (PYY), leptin and ghrelin were determined by multiplex immunoanalyses based on the xMAP technology using MILLIPLEX MAP Human Gut Hormone Panel (Millipore, Billerica, MA, USA) and Luminex 100 IS instrument (Luminex Corporation, Austin, USA).

Oxidative stress markers: The amount of lipid peroxidation was determined as thiobarbituric acid reactive substances (TBARS) using a modified method according to Yokode [13]. The activity of superoxide dismutase (SOD) was analyzed by superoxide dismutase assay kit (Cayman Chemical, MI, USA). The serum level of ascorbic acid was measured by the spectrophotometric method as previously described [14]. The whole blood level of reduced glutathione was determined with the Glutathione HPLC diagnostic kit (Chromsystems, Munich, Germany).

### Statistical analyses

For statistical analysis, repeated-measures ANOVA was used. The factors of group, subject and time were included in the model. Interactions between group and time (group×time) were calculated for each variable. Within each group, paired comparison t-tests were calculated to test whether the changes from baseline to 30′, from 30′ to 60′, from 60′ to 120′ and from 120′ to 180′ were statistically significant. Pearson correlations were calculated for the relationship between changes in oxidative stress markers and changes in gastrointestinal hormones. Data are presented as mean with 95% CI.

## Results

**Figure 1:**
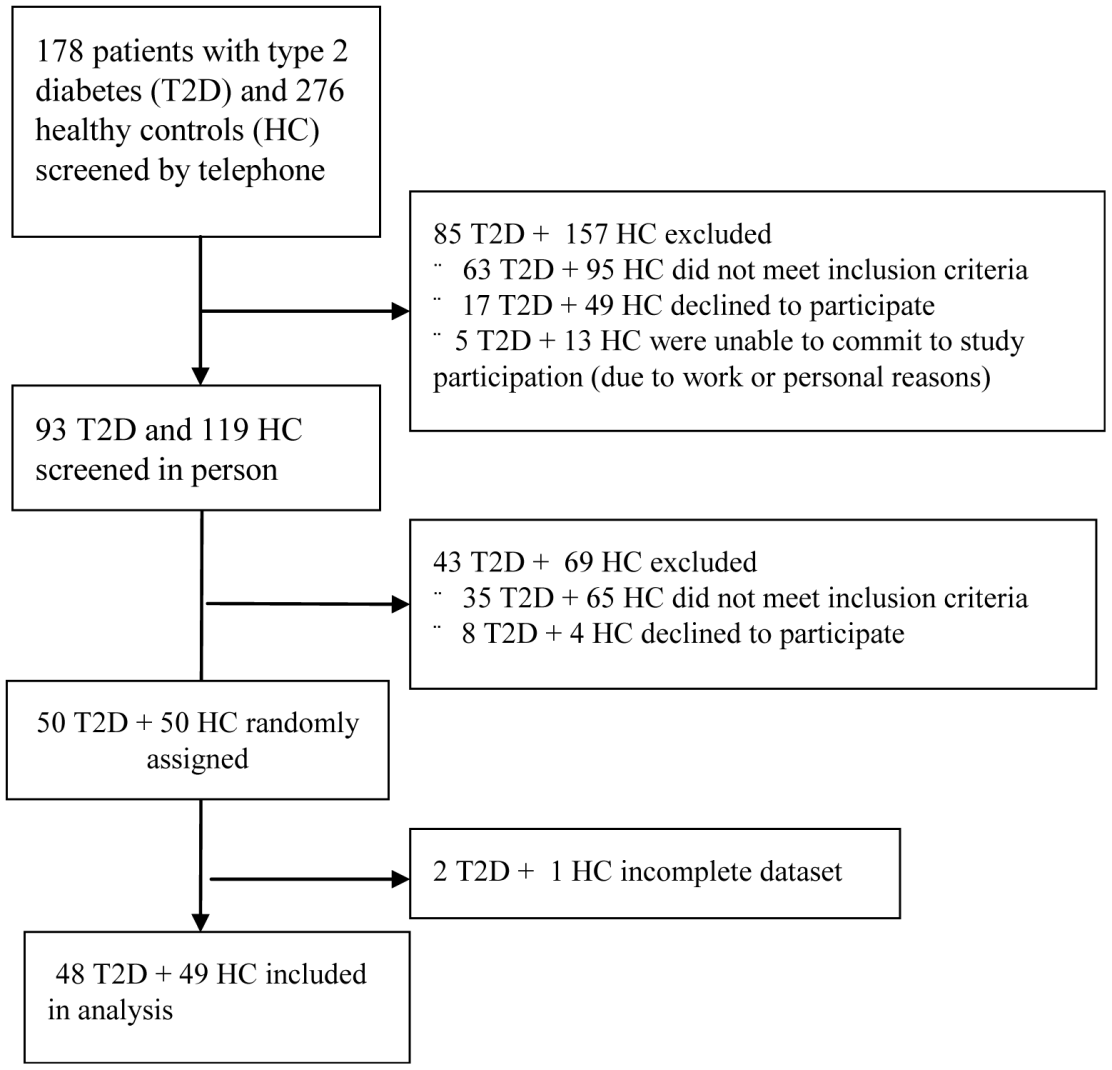
Enrollment of the participants and completion of the study.

The number of participants included and dates defining the periods of recruitment and follow-up are shown in Figure 1. The authors have not observed harms or unintended effects of consumed meals of any kind in participants.

**Figure 2:**
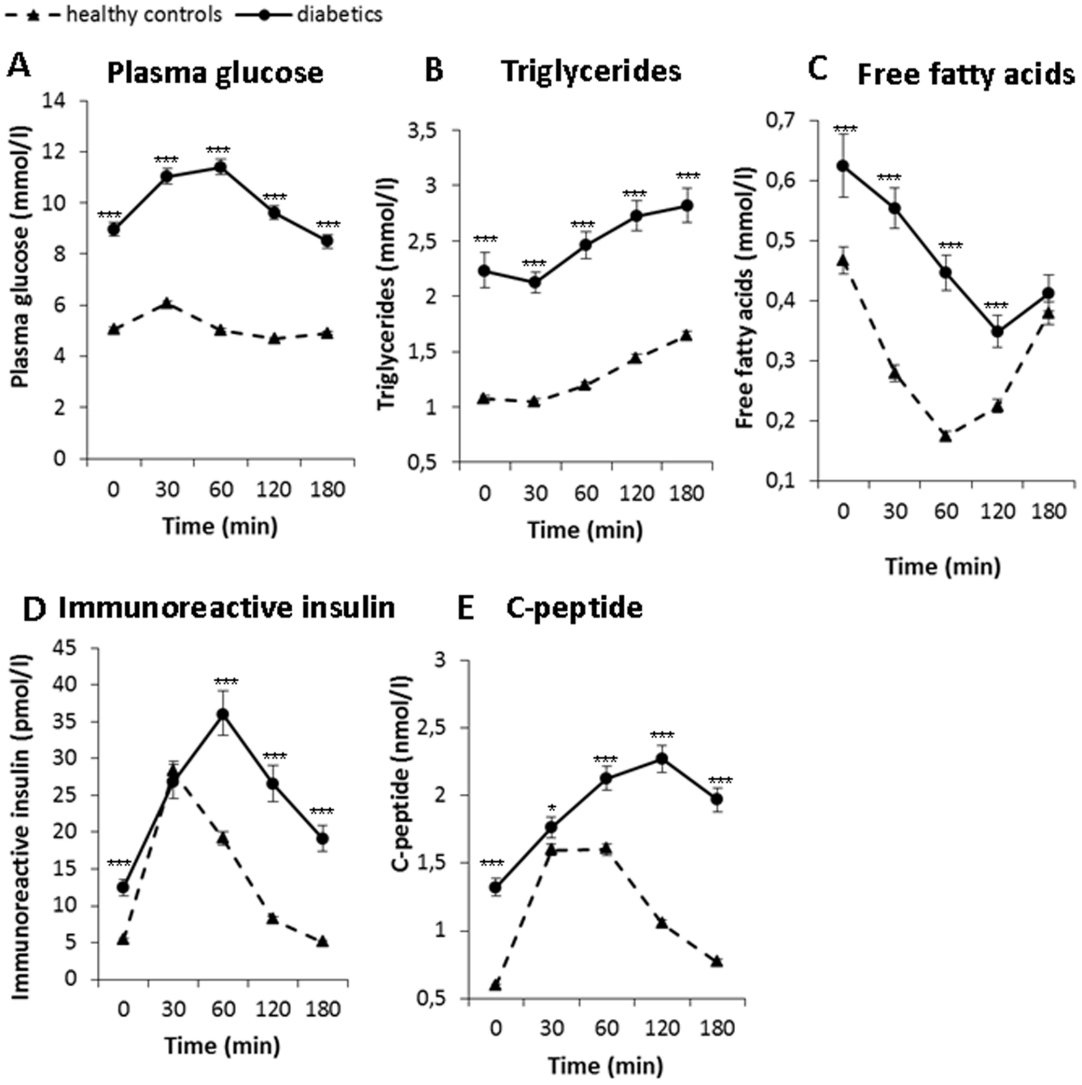
Postprandial changes in plasma concentrations of glucose, lipids and insulin in diabetic (circles, full line) (n = 48) and control subjects (triangles, dashed line) (n = 49) after the standard meal test. Data are expressed as mean with 95% CI. A: Plasma glucose: Factors time p<0.001, group p<0.001, interaction group×time p<0.001, B: Triglycerides: Factors time p<0.001, group p<0.001; interaction group×time p = 0.001, C: Free fatty acids: Factors time p<0.001, group p<0.001, interaction group×time p<0.001, D: Immunoreactive insulin: Factors time p<0.001, group p<0.001, interaction group×time p<0.001, E: C-peptide: Factors time p<0.001, group p<0.001, interaction group×time p<0.001.

The plasma concentrations of glucose, lipids, IRI and C-peptide in fasting and postprandial state after the standard meal test are illustrated in Figure 2. All these measured parameters were significantly higher in diabetic subjects than in healthy controls at virtually every time point after the standard meal. Plasma concentrations of triglycerides were inversely related to plasma concentrations of free fatty acids in both diabetic and healthy subjects.

### Gastrointestinal hormones

**Figure 3:**
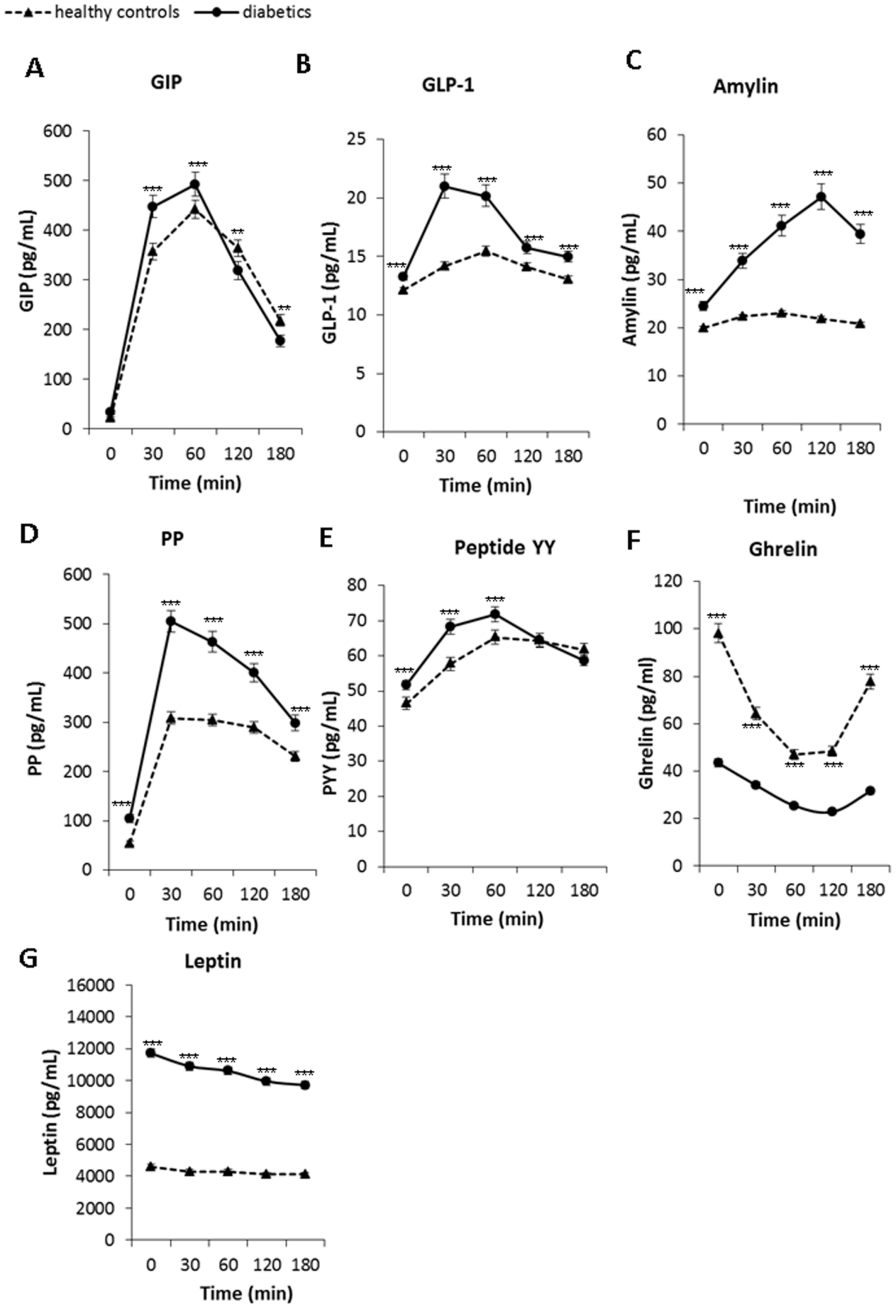
Postprandial changes in plasma concentrations of gastrointestinal hormones in diabetic (circles, full line) (n = 48) and control subjects (triangles, dashed line) (n = 49) after the standard meal test. Data are expressed as mean with 95% CI. A: – GIP: Factors time p<0.001, group p<0.001, interaction group×time p<0.001, B: GLP-1: Factors time p<0.001, group p<0.001, interaction group×time p<0.001, C: amylin: Factors time p<0.001, group p<0.001, interaction group×time p<0.001, D: PP: Factors time p<0.001, group p<0.001, interaction group×time p = 0.001, E: PYY: Factors time p<0.001, group p<0.001, interaction group×time p = 0.002, F: Ghrelin: Factors time p<0.001, group p<0.001, interaction group×time p<0.001 and G: Leptin: Factors time p<0.001, group p<0.001, interaction group×time p = 0.2.

Both basal and postprandial concentrations of almost all gastrointestinal hormones were significantly higher in patients with T2D compared to healthy controls (see Figure 3). The most notable differences between diabetics and healthy controls were observed in postprandial secretion of amylin, GLP and PP, in both quantity as well as dynamics (Figure 3). However, there were differences in dynamics between individual gastrointestinal peptides. The increase in postprandial secretion of GLP and PP was rapid, the maxium peak in postmeal phase was observed after 30 min. On the other hand, the postprandial secretion of amylin increased slowly, the maximum peak in postmeal phase of amylin was observed after 120 min. The postmeal dynamics of GIP secretion was strong, however differences between patients with T2D and healthy controls were not as pronounced as in GLP. The lowest occurrence of postprandial changes was observed in secretion of PYY.

### Appetite hormones

The concentrations of ghrelin and leptin differ significantly between patients with T2D and healthy controls during the whole meal test as shown in Figure 3 (F and G). In the fasting state, plasma concentrations of ghrelin were lower in diabetic subjects by 56% and plasma concentrations of leptin were elevated by 150% compared to healthy controls. Plasma concentrations of ghrelin were significantly lower and those of leptin significantly higher in patients with T2D during the whole meal test. The physiological postprandial suppression of ghrelin secretion was not as much notable in diabetic subjects as in healthy controls (see Figure 3). Despite the significant increase of leptin concentrations in patients with T2D, the postprandial dynamics of leptin was slightly notable in both groups.

### Oxidative stress parameters

**Figure 4:**
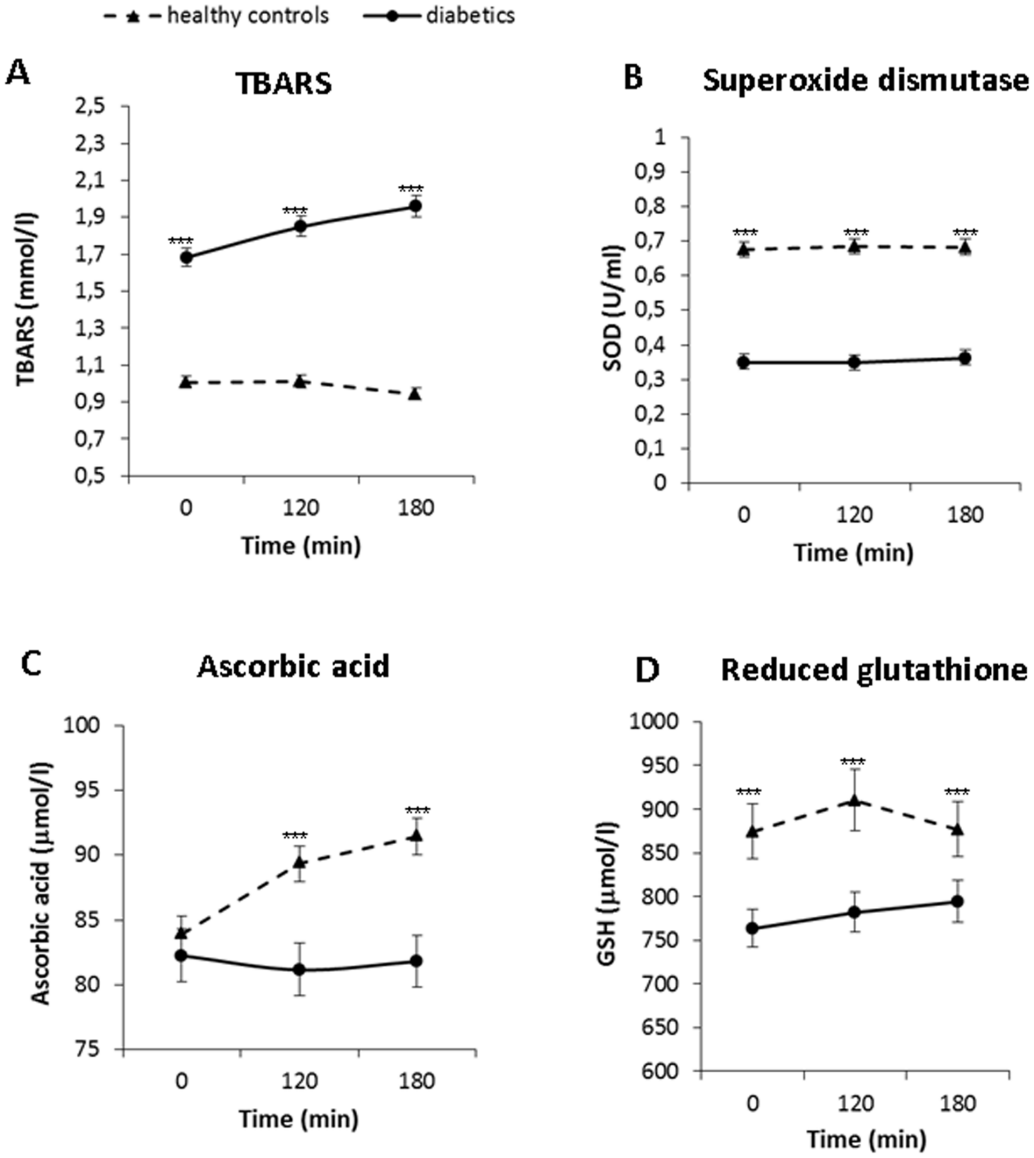
Postprandial changes in plasma concentrations of oxidative stress markers in diabetic (circles, full line) (n = 45) and control subjects (triangles, dashed line) (n = 49) after the standard meal test. Data are expressed as mean with 95% CI. A: TBARS: Factors time p<0.05, group p<0.001, interaction group×time p<0.01, B: SOD: Factors time p = 0.7, group p<0.001, interaction group×time p = 0.13, C: Ascorbic acid: Factors time p = 0.55, group p<0.001, interaction group×time p<0.01, D: GSH: Factors time p = 0.33, group p<0.001, interaction group×time p = 0.67.

In basal conditions (time 0) all of the measured oxidative stress markers were different in patients with T2D compared to control subjects: TBARS were increased by 67% while ascorbic acid, reduced glutathione and SOD activity were decreased in diabetic subjects by 5%, 13% and 48%, respectively (Figure 4).

Lipid peroxidation measured as TBARS increased during the postprandial phase together with the changes in plasma glucose and triglycerides in diabetic patients (P<0.001). In healthy controls no postprandial dynamics of TBARS was observed (Figure 4). There were no significant changes in plasma concentrations of ascorbic acid during the postprandial phase in diabetic patients, while in healthy controls ascorbic acid increased (P<0.01; Figure 4). Plasma concentrations of reduced glutathione or superoxide dismutase activity did not change significantly either in diabetics or in healthy controls.

### Correlations

Postprandial secretion of measured gastrointestinal hormones was increased in parallel with glucose and insulin concentrations in patients with T2D. As shown in table 2, a positive relationship was found between Δ GIP and Δ glucose and Δ IRI, between Δ PYY and Δ glucose, and between Δ amylin and Δ IRI and Δ C-peptide in patients with T2D. No significant relationship was observed between the changes in any gastrointestinal hormones and the changes in glucose or insulin in healthy controls (data not shown). Changes in triglycerides correlated negatively with Δ PP and Δ ghrelin and positively with Δ amylin. Changes in ascorbic acid correlated positively with Δ GIP and Δ PYY in diabetic patients.

**Table 2:** Correlation between changes in gastrointestinal hormones, leptin and ghrelin and changes in oxidative stress and metabolic parameters in patients with T2D (n = 45).

	Δ glu	Δ IRI	Δ C-pep	Δ Tg	Δ FFA	Δ AA	Δ TBARS	Δ SOD	Δ GSH
**Δ GIP**	**0.5196** ***	**0.5420** ***	−0.0215	0.0570	0.3295	**0.2702** *	−0.0933	−0.2453	0.2028
**Δ GLP**	0.2191	0.0943	0.0034	−0.0175	0.2129	−0.1234	−0.1929	0.0326	0.0933
**Δ PP**	0.0981	−0.0720	−0.2725	**−0.4852** **	**0.2872** *	0.1191	−0.0871	0.0668	−0.1936
**Δ PYY**	**0.4661** **	0.1033	−0.0216	−0.1600	0.0887	**0.5293** ***	−0.0347	−0.0091	−0.3344
**Δ amylin**	−0.0708	**0.6819** ***	**0.7336** ***	**0.3130** *	−0.1229	−0.1800	0.0505	−0.1698	0.0616
**Δ ghrelin**	0.0568	−0.2784	−0.2553	**−0.2972** *	0.0309	−0.2903	0.2310	0.3146	−0.0596
**Δ leptin**	0.1537	0.0958	0.0269	−0.1352	−0.0097	−0.0456	0.2907	−0.1908	−0.1528

*denote p<0.05,

**denote p<0.01,

***denote p<0.001.

plasma glucose (glu), immunoreactive insulin (IRI), C-peptide (C-pep), triglycerides (Tg), free fatty acids (FFA), ascorbic acid (AA), TBARS, superoxide dismutase (SOD) and reduced glutathione (GSH).

## Discussion

In the study in question the authors monitored postmeal response of gastrointestinal hormones and oxidative stress markers in diabetic patients and compared them with healthy controls. The postmeal phase is an important and independent predictor of macrovascular diabetic complications, more in females than in males [15]. Postprandial hyperglycemia is a stronger cardiovascular risk factor in women than in men, whereas other authors state that gender-related differences disappear after adjustment for the main cardiovascular risk factors [15]. In our study we observed the postprandial glycemic control in the general population and the proportion of women and men was equal.

Elevation of postmeal or postchallenge glucose supports the concept of “metabolic memory” [9] which is responsible for early diabetic complications and which is closely tied to oxidative stress, namely with increased mitochondrial superoxide production.

However, few studies were interested in postprandial phase after a meal test, which is more physiological as it contains all main nutrients than the usually used oral glucose tolerance test.

According to Alssema study [18], incretin effect could be distinct after OGTT and after a standard meal test. In this study GLP-1 secretion in diabetic patients was increased following oral glucose but not after the mixed meal [18]. Therefore, incretin secretion seems to depend on both the glucose and lipid metabolism as well.

The incretin effect is diminished secondarily in T2D as a concequence of metabolic and hormonal disturbances [16], [17] while increased oxidative stress is directly involved in the pathogenesis of diabetes [26]. The authors focused on clarifying whether these parameters correlated with each other and whether they had mutual influence on each other.

Several studies have shown that the incretin effect is attenuated in T2D because of a severe defect in β-cell sensitivity to GIP [5], [6], which has an insulinotropic effect [19]. It has also been suggested that changes in insulin secretion following a lifestyle intervention might be mediated via alterations in GIP secretion [20].

GIP, secreted strongly in response to fat ingestion, is involved in the translation of excessive amounts of dietary fat into adipocyte tissue stores [21]. Patients with T2D are resistant to the biological effects of GIP [22]. Specific GIP receptor antagonists improve glucose tolerance and β-cell function by amelioration of insulin resistance in *ob/ob* mice [23]. These effects are similar to improvements of metabolism after bariatric surgery in humans [24]. The blockade of GIP action appears promising as a new and potentially important approach to treat obesity-related diabetes [25].

PYY is released postprandially from gastrointestinal L-cells with GLP-1 and oxyntomodulin [28] and has anorexic effects [29]. In healthy humans stimulation of PYY and PP is dependent on fat digestion [30]. In obese subjects, the altered postprandial secretion of PYY is a consequence of a dysfunction of L cells, which become less sensitive to the positive feedback effect of lipids [31].

The positive correlation of changes in amylin, insulin and C-peptide observed by the authors is not surprising. Amylin is a peptide co-secreted with insulin. The role of amylin in the pathogenesis of T2D has been suggested by in vitro and in vivo studies indicating its effect to cause insulin resistance and/or inhibit insulin secretion [32]. It is worth noting that amylin interacts with numerous other gastrointestinal hormones to control eating and mediate the eating inhibitory effect of some of these hormones, most prominently peptide YY and GLP-1 [27]. These combinations lead to a stronger reduction of eating control than single hormones alone. Thus the diminished effect of amylin is possibly important for other gastrointestinal hormones.The positive correlation between postprandial changes in amylin and triglycerides is in accordance with a study which demonstrated a strong association of amylin with inflammatory markers and metabolic syndrome including triglycerides in healthy individuals [33].

On the other hand, postprandial changes in PP associated negatively with triglycerides changes and positively with FFA changes in patients with T2D. As suggested earlier, elevated plasma PP may be viewed as a negative marker and it has been demonstrated that after diet-induced weight loss, the decrease in PP correlated negatively with improvement in β-cell function [34]. To the best knowledge of the authors, the association between PP and postprandial lipids has not been published yet.

We observed lower fasting and postprandial plasma ghrelin and diminished postprandial suppression of ghrelin secretion in patients with T2D. That is in accordance with the previously demonstrated lower concentrations of ghrelin in response to weight gain, overfeeding and a high-fat diet [35]. Metformin prolongs the postprandial fall in ghrelin concentrations in patients with T2D, which is one of its potential mechanisms of promoting weight loss [36].

A negative association was found between postprandial changes in ghrelin and in triglycerides. Although the authors of this study are the first ones to demonstrate a direct association between these variables, there is already some evidence in the literature supporting their finding: It has been demonstrated that a high-fructose diet attenuates postprandial suppression of ghrelin and increases triglycerides in healthy women, however the association has not been tested by the authors [37]. One experimental study demonstrated that ghrelin administration lowers muscle triglycerides in rat muscle [38].

As a new finding the authors observed a positive relationship of postprandial changes in GIP and PYY with changes in ascorbic acid in patients with T2D. The correlation does not prove any causal relationship. Either the primary defect is the dysfunction of L and K cells of the intestine, resulting in abnormalities in postprandial plasma glucose and lipids and causing an increased oxidative stress, or the primary defect is the increased postprandial oxidative stress due to hyperglycemia and hyperlipidemia, causing a dysfunction of L and K cells. There is also the option that both abnormalities go hand in hand with no causal relationship. In this study diabetic patients have distinct postmeal dynamics of oxidative stress parameters compared to healthy controls. However, the postmeal response is lower than that of gastrointestinal hormones. It has been shown that glucose and ascorbic acid compete for entry into the cells, so that postprandial increases in glucose inhibit the input of ascorbic acid to the cells [39]. Postprandial hyperlipidemia prolonged endothelial dysfunction and ascorbic acid is able to improve endothelial dysfunction and attenuates the oxidative stress induced by postprandial lipids [40]. However, no relationship between ascorbic acid and ganstrointestinal hormones has been described yet. The parameters of oxidative stress could also be affected by smoking habit. In our study the proportion of smokers is relatively low and the ratio is almost identical in both groups. In each subject we observed the individual postprandial response and therefore each subject was evaluated in relation to individual basal conditions. This is the reason why we did not separate smokers and non-smokers.

The strength of this study is that the authors used physiological stimulation by a standard mixed meal, where insulin secretory responses are related to the incretin axis which allowed the authors to study the secretion of gastroinestinal hormones during a physiological postprandial pertubation. A potential weakness of the study is that immunoanalytical methods used to determine the concentrations of gastrointestinal peptides are accurate to ±10–20%. The difference in fasting plasma concentrations of GLP-1 and PYY between diabetic subjects and healthy controls does not exceed the accuracy of the method. Furthermore, diabetic patients had a significantly higher body weight and BMI compared to the control subjects and this could affect some of the responses reported. In healthy subjects no effect of adiposity on postprandial GIP and GLP-1 levels was observed [41]. The effect of obesity on other gastrointestinal hormones response is not clear.

In conclusion, the results proved impaired basal and postprandial secretion of gastrointestinal hormones in patients with type 2 diabetes as well as increased postprandial oxidative stress compared to healthy controls. Besides a positive relationship of postprandial changes in GIP and PYY with changes in ascorbic acid, there was no direct link between gastrointestinal hormones and oxidative stress markers in patients with T2D. Diminished effect of gastrointestinal hormones and increased oxidative stress are, probably, two independent mechanisms in diabetes and it should be considered in therapeutical approach.

## Supporting Information

Checklist S1 **CONSORT Checklist.**
(DOC)

Protocol S1 **Trial Protocol.**
(DOC)
