# Supplementary material for: Postprandial Oxidative Stress and Gastrointestinal Hormones: Is There a Link?
Source: PLoS One. 2014 Aug 20;9(8):e103565. doi: 10.1371/journal.pone.0103565 (PMC4139261; doi:10.1371/journal.pone.0103565)
Supplement: Protocol S1 — Trial Protocol. (DOC) [file pone.0103565.s002.doc]

**Postprandial Insulin Secretion, Oxidative Stress and Gastrointestinal (GI) Peptides in Patients With Type 2 Diabetes Versus Healthy Subjects**

**Introduction:**

Postprandial state in patients with type 2 diabetes mellitus is characterized by increased oxidative stress markers and changes in the secretin of gastrointestinal peptides.

**Aims and priorities of the project:**

The purpose of this study is to

1. compare fasting and post-load blood glucose excursions, insulin secretion, oxidative stress markers and GI peptide release from the GI tract in individuals with type 2 diabetes and healthy controls after three different meals with the same caloric content but different composition
2. characterize some of the mechanisms of action of a vegetarian meal in regards to secretion of GLP-1, amylin and other GI peptides (GIP, glucagon, ghrelin, PYY and PP) and in regards to β-cell function

**Hypothesis:**

Vegetarian meal will lead to increased secretion of gastrointenstinal peptides and decreased secretion of oxidative stress markers

**Methods:**

A randomized study on 60 patients with type 2 diabetes and 60 healthy controls.

***Study group:***

1. **60 patients with T2D**

Inclusion criteria:

1. Type 2 diabetes mellitus for at least one year
2. Treatment of T2D: diet or oral antidiabetic agents (stable drug therapy at least 3 month before the trial
3. The presence of metabolic syndrome – any three of the following symptoms:
   1. Abdominal obesity – waist circumf. in men> 102 cm, in women > 88 cm
   2. Diagnosis and treatment of type 2 diabetes or raised fasting plasma glucose level (FPG> 5,6 mmol/l)
   3. Raised blood pressure (BP): systolic BP > 130 mm Hg or diastolic BP >85 mm Hg, or treatment of previously diagnosed hypertension
   4. Reduced HDL cholesterol in men < 1 mmol/l, in women < 1,3 mmol/l (or treatment)
   5. Raised triglycerides > 1,7 mmol/l (or treatment)
4. HbA1c (according to IFCC) ≥4.2 a ≤10.5%
5. Men and women aged 30-65 years
6. Body Mass Index (kg/m^2^) in the range of 27 – 50

Exclusion criteria:

1. Type 1 diabetes mellitus
2. Unstable drug therapy at least 3 month before the trial
3. Treatment with Byetta or Victosa
4. Pregnancy (positive β-HCG test), breast feeding or trying to become pregnant
5. Alcoholism or drug use
6. Presence of other medical condition, which occurs during physical examination, laboratory tests, ECG, including pulmonary, neurological or inflammatory disease, which would be considered by the examiner to distort the consistency of data
7. **Healthy subjects:**

Inclusion criteria:

1. Men and women aged 30-65 years
2. Body Mass Index (kg/m2) less than 30
3. Absence of metabolic syndrome – not more than any two of the following symptoms:
4. Abdominal obesity – waist circumf. in men> 102 cm, in women > 88 cm
5. Diagnosis and treatment of type 2 diabetes or raised fasting plasma glucose level (FPG> 5,6 mmol/l)
6. Raised blood pressure (BP): systolic BP > 130 mm Hg or diastolic BP >85 mm Hg, or treatment of previously diagnosed hypertension
7. Reduced HDL cholesterol in men < 1 mmol/l, in women < 1,3 mmol/l (or treatment)
8. Raised triglycerides > 1,7 mmol/l (or treatment)

1. Absence of diabetes in first- line relatives

Exclusion criteria:

1. Unstable drug therapy at least 3 month before the trial
2. Pregnancy (positive β-HCG test), breast feeding or trying to become pregnant
3. Alcoholism or drug use
4. Presence of other medical condition, which occurs during physical examination, laboratory tests, ECG, including pulmonary, neurological or inflammatory disease, which would be considered by the examiner to distort the consistency of data

**Flowchart:**

**Visit 1: Screening → Randomization → Visit 2: meal test 1 → Visit 3: meal test 2→ Visit 4: meal test 3**

**Visit 1: Screening:**

Common anthropometric investigations: body weight, BMI, waist and hip circumferences, blood pressure

Basic blood tests: Na, K, Cl, urea, creatinine, bilirubin, transaminases, HbA1c, HIV, HbsAg, cholesterol, triglycerides, HDL cholesterol, LDLcholesterol, plasma glucose, CBC

The subjects will be explained aims, methods and risks of the study and they will sign informed consent (Appendix 1).

**Visit 2/3/4: Meal tests**

Plasma glucose, IRI, plasma lipids, oxidative stress markers and gastrointestinal peptides measured in response to 3 meals (at times 0', 30', 60', 120' and 180')

Study participants arrive at 7 – 8 in the morning, fasting (8 - 12 hours). The night and morning before the procedure, patients with diabetes do not take medication for diabetes. Participants will complete the questionnaires.

***Meal composition:***

**1. Crocodille Baguette Cheese Gourmet** *(a conventional diabetic meal)*

Weight 180 g, energy 452,8 Kcal/1895,7 kJ, composition: carbohydrates 49,2 g (44,55%), proteins 18,5 g (16,74%), lipids 18,8 g (38,7%), from which saturated fatty acids are 6,8 g, monounsaturated are 6,0 g, polyunsaturated are 5,0 g and fiber is 5 g.

**2. Couscous burger** *(a vegan meal)*

Weight 235 g, energy 459 Kcal /1918 kJ, composition: carbohydrates 56,34 g (49,1%), proteins 12,03 g (10,48% %), lipids 20,5 g (40,2%), from which saturated fatty acids are 2,31 g, monounsaturated are 5,49 g, polyunsaturated are 10,5 g and fiber is 7,74 g.

**3. McCountry sandwich** *(a meal rich in saturated fats)*

Weight 150 g, energy 455 kcal/1903,72 kJ, composition: carbohydrates 31 g (27,25%), proteins 24 g (21,1%), lipids 26 g (51,43%), from which saturated fatty acids are 10 g, monounsaturated are 6,0 g, polyunsaturated are 8,0 g and fiber is 5 g.

**Blood samples:**

1. Plasma glucose – 0, 30, 60, 120 a 180 min.
2. Immunoreactive insulin – 0, 30, 60, 120 a 180 min.
3. C-peptide– 0, 30, 60, 120 a 180 min.
4. Triglycerides, free fatty acids – 0, 30, 60, 120 a 180 min.
5. Gastrointestinal peptides (leptin, ghrelin, GLP-1, GIP, PPY, PP, amylin) - test tube with inhibitors (DPP-IV inh., Protease inh., Pefabloc) – 0, 30, 60, 120 a 180 min.
6. Oxidative stress markers (SOD, CAT, GPx, GR, GST, αE, γE, AA, GSH, TBARS, CD) - 0,120,180 min
7. 2x stock serum - 0, 30, 60, 120 a 180 min.

**Appendix 1: Information for patients and informed consent**

**Postprandial Insulin Secretion, Oxidative Stress and Gastrointestinal (GI) Peptides in Patients With Type 2 Diabetes Versus Healthy Subjects**

The purpose of this study is to compare fasting and post-load blood glucose excursions, insulin secretion and peptide release from the GI tract in 60 individuals with type 2 diabetes and in 60 healthy controls after three different meals with the same caloric content but different composition. To participate in the study means to come for three meal tests in cca 1-week-intervals.

Each meal test which will take about 3,5 hours - we will take a blood sample, then give you breakfast and take other blood samples after 30, 60, 120 and 180 minutes after the meal. The physician will also examine you and answer your questions. You will eat three sandwiches: 1. Crocodille baguette Cheese Gourmet (a conventional diabetic meal), 2. Couscous burger (a vegetarian meal), and 3. McCountry sandwich (a meal rich in saturated fats).

All the above explained investigations do not make up any risk There is no testing of new drugs. All of the above are functional investigations that will not result in treatment changes. You can withdraw from our study whenever you want to without giving reason for doing so and without consequences for your further treatment. All documents related to the study will be anonymous and will be archived.

I have read and understood all the above information regarding the study and I have had the opportunity to discuss it with Dr...............

I agree to participate in this study.

Name ______________________

Signature __________________
